# Comprehensive bioinformatics analysis reveals the significance of forkhead box family members in pancreatic adenocarcinoma

**DOI:** 10.18632/aging.204455

**Published:** 2023-01-04

**Authors:** Wei Hu, Mingxu Li, Yan Wang, Chengcheng Zhong, Xinxin Si, Xiao Shi, Zhong Wang

**Affiliations:** 1 Department of Hepatobiliary Surgery, Affiliated Lianyungang Hospital of Xuzhou Medical University, Lianyungang 222001, Jiangsu, P.R. China; 2 Department of Pathology, The Second People’s Hospital of Lianyungang, Lianyungang 222001, Jiangsu, P.R. China; 3 Jiangsu Key Laboratory of Marine Pharmaceutical Compound Screening, College of Pharmacy, Jiangsu Ocean University, Lianyungang 222005, Jiangsu, P.R. China

**Keywords:** forkhead box family, pancreatic adenocarcinoma, biomarker, prognosis, senescence

## Abstract

Background: Forkhead box proteins (FOXs) play important roles in multiple biological processes; while little is known regarding the role of FOX members in pancreatic adenocarcinoma (PAAD). This study aimed to comprehensively investigate the function of FOX family members in PAAD.

Methods: Expression and prognostic value of FOXs were analyzed by R language and GEPIA. Genetic alteration and promoter methylation level were analyzed using CBioPortal and UALCAN. Protein-protein interactions and gene functions were analyzed using STRING and DAVID. TIMER and SENESCopedia were utilized to analyze the correlation of FOXs with immune cell infiltration or tumor senescence. Protein levels of FOXs were detected by immunohistochemistry.

Results: Expression of 15 of 50 FOXs were significantly elevated in PAAD. Among these 15 differentially expressed FOXs (DE-FOXs), 4 were significantly associated with the clinical cancer stage and 4 were negatively associated with overall survival. Functions of DE-FOXs were related to epithelial tube morphogenesis, nuclear chromatin, and DNA-binding. Promoter methylation and genomic alterations were not major causes of FOX dysregulation. Most DE-FOX was correlated with diverse immune infiltration cells. Seven of the DE-FOXs were positively related to tumor senescence. The protein levels of FOXM1, FOXP1, and FOXN3 were negatively correlated with OS in the collected PAAD patients.

Conclusions: FOXM1, FOXP1, and FOXN3 have prognostic value. Seven FOXs were related senescence, whereas most DE-FOXs were related to immune infiltration in PAAD. Our findings are instructive for future research on FOX family and provide novel insights into the selection of FOXs with potential prognostic or therapeutic target value.

## INTRODUCTION

Pancreatic adenocarcinoma (PAAD) is characterized by late diagnosis, low treatment success rates, and poor survival prognosis [1]. The most common and effective treatment therapy is surgical excision, but most patients are diagnosed at an advanced stage, making surgical resection difficult. Consequently, chemotherapy agents, such as gemcitabine plus nab-paclitaxel, have been used to improve patient survival [2]. However, due to complex biology or heterogeneity, PAAD patients show poor chemotherapeutic responses or therapeutic resistance [3]. Inhibiting the mechanism(s) that promote therapy resistance and targeting the key pathways essential for PAAD survival may therefore improve patient outcomes, while seeking new therapeutic targets may deliver better clinical PAAD outcomes or overcome existing resistance.

The forkhead box (FOX) family of transcription factors play important roles in regulating the expression of genes involved in cell growth, proliferation, differentiation, apoptosis, immune function, and longevity. The homology degree of the forkhead domains classifies the 50 coding genes of FOX family into 19 subfamilies (FOXA to FOXS) in humans [4], and these family members display diverse functions in differentiation, apoptosis, and immunity. Many FOXs, such as the FOXA, FOXO, and FOXI subfamilies, are known as pioneer factors that regulate the accessibility of other transcription factors to chromatin.

The FOXs in various tumors show gene amplification, deletion, chromosomal translocation, mutation, and fusion. For example, as a result of gene amplification, FOXM1 is abnormally high expressed in basal-type breast cancer and diffuse large B-cell lymphoma [5]. Dysregulated expression of FOX genes also occurs in human cancers. Notably, the function of FOXs may have organizational diversity, as a decrease in FOXA1 expression is associated with tumor infiltration in bladder cancer, whereas overexpression of FOXA1 is associated with good prognosis in triple negative breast cancers [6, 7]. Consequently, the role of the FOX family in cancer has attracted widespread attention. However, systematic studies are lacking regarding the functions of FOX family members in pancreatic adenocarcinoma (PAAD).

The aim of the present study was to use The Cancer Genome Atlas (TCGA) and Gene Expression Omnibus (GEO) datasets, the GEPIA, cBioPortal, UALCAN, STRING, and TIMER online analysis websites, and R language to conduct a comprehensive investigation of the functions of FOX family members. Our findings extend knowledge about the expression and potential clinical value of distinct FOXs and the possible relationship between FOXs and immune cell infiltration or tumor senescence in PAAD.

## RESULTS

### Differential expression of FOXs in PAAD patients

**Figure 1:**
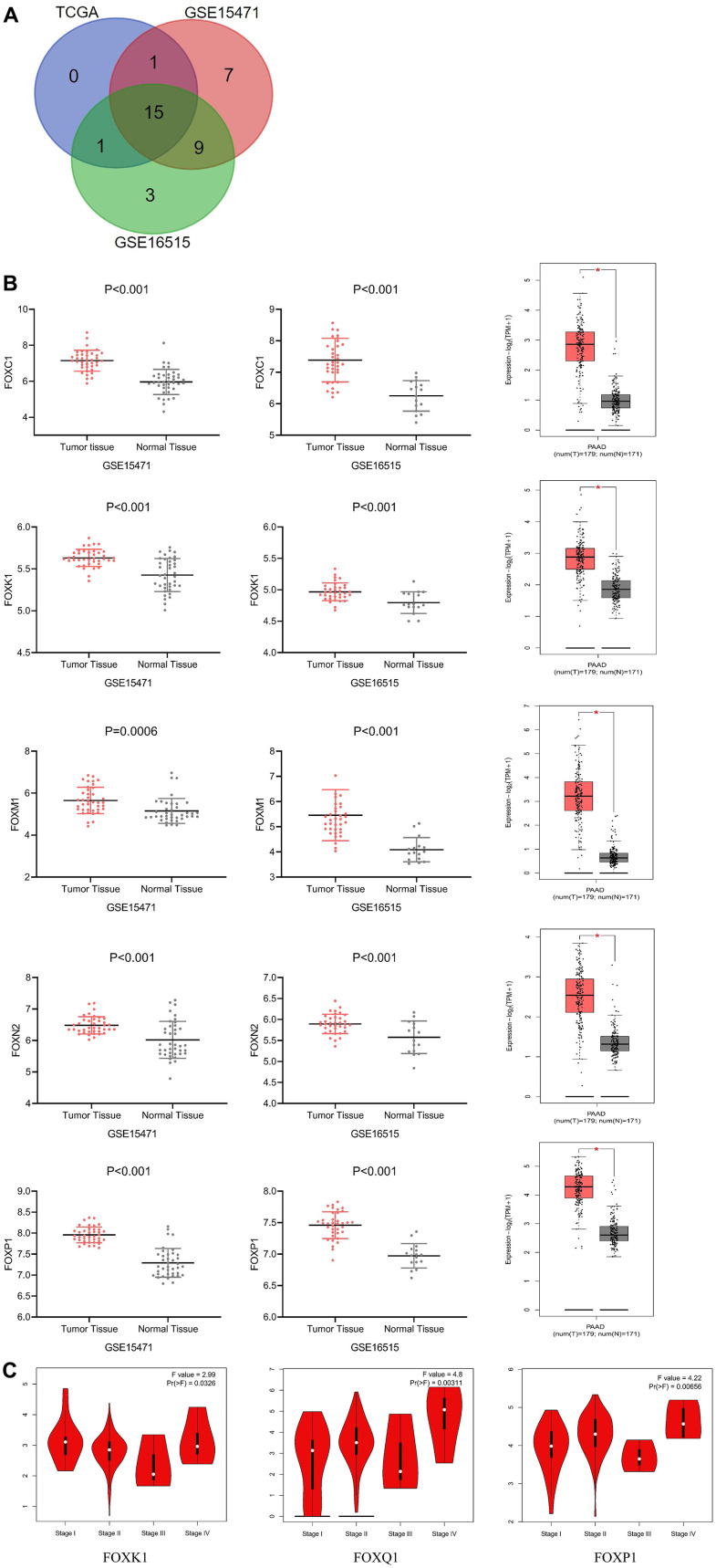
**Differential expression and correlation with tumor stages of FOXs in PAAD patients.** (**A**) Differentially expressed genes were selected with P < 0.01 and |Log2FC| Cutoff ≥1 among the datasets GSE15471, GSE16515, and TCGA. The Venn diagram shows an overlap of 15 genes that had higher expression in cancer tissues than in adjacent tissues. (**B**) Expression of representative FOXs in PAAD (GSE15471, GSE16515 and GEPIA). GSE15471: number(normal)=39, number(tumor)=39; GSE16515: number(normal)=36, number(tumor)=16. (**C**) Correlations between FOX expression and tumor stage in PAAD patients (TCGA). The expression of FOXK1, FOXQ1, and FOXP1 was correlated with the pathological stage of PAAD patients (p < 0.05). PAAD: pancreatic adenocarcinoma.

We conducted mRNA analysis to explore the expression of different FOXs in PAAD tumor and normal tissues. We chose three different PAAD patient RNA sequencing data sets (GSE15471, GSE16515, and TCGA) for the analysis, and we screened out potential prognostic markers of PAAD based on higher expression of FOX genes in cancer than in normal tissues. The three data sets revealed 15 FOX members whose expression in cancer tissues were all elevated (Figure 1A): FOXA1, FOXC1, FOXC2, FOXD1, FOXF1, FOXF2, FOXJ1, FOXK1, FOXL1, FOXM1, FOXN2, FOXN3, FOXP1, FOXQ1, and FOXS1. Figure 1B shows the differential expression of 5 representative genes (FOXC1, FOXK1, FOXM1, FOXN2, and FOXP1) from the three data sets.

GEPIA assessment of the correlation between expression of the 15 differentially expressed FOXs (DE-FOXs) in the TCGA PAAD data set and the pathological stage of PAAD patients revealed a significant association between FOXK1, FOXQ1, and FOXP1 and tumor stages (Figure 1C). The expression of other FOXs did not differ markedly with pathological stage (data not shown).

### Prognostic value of the expression of FOXs in PAAD patients

**Figure 2:**
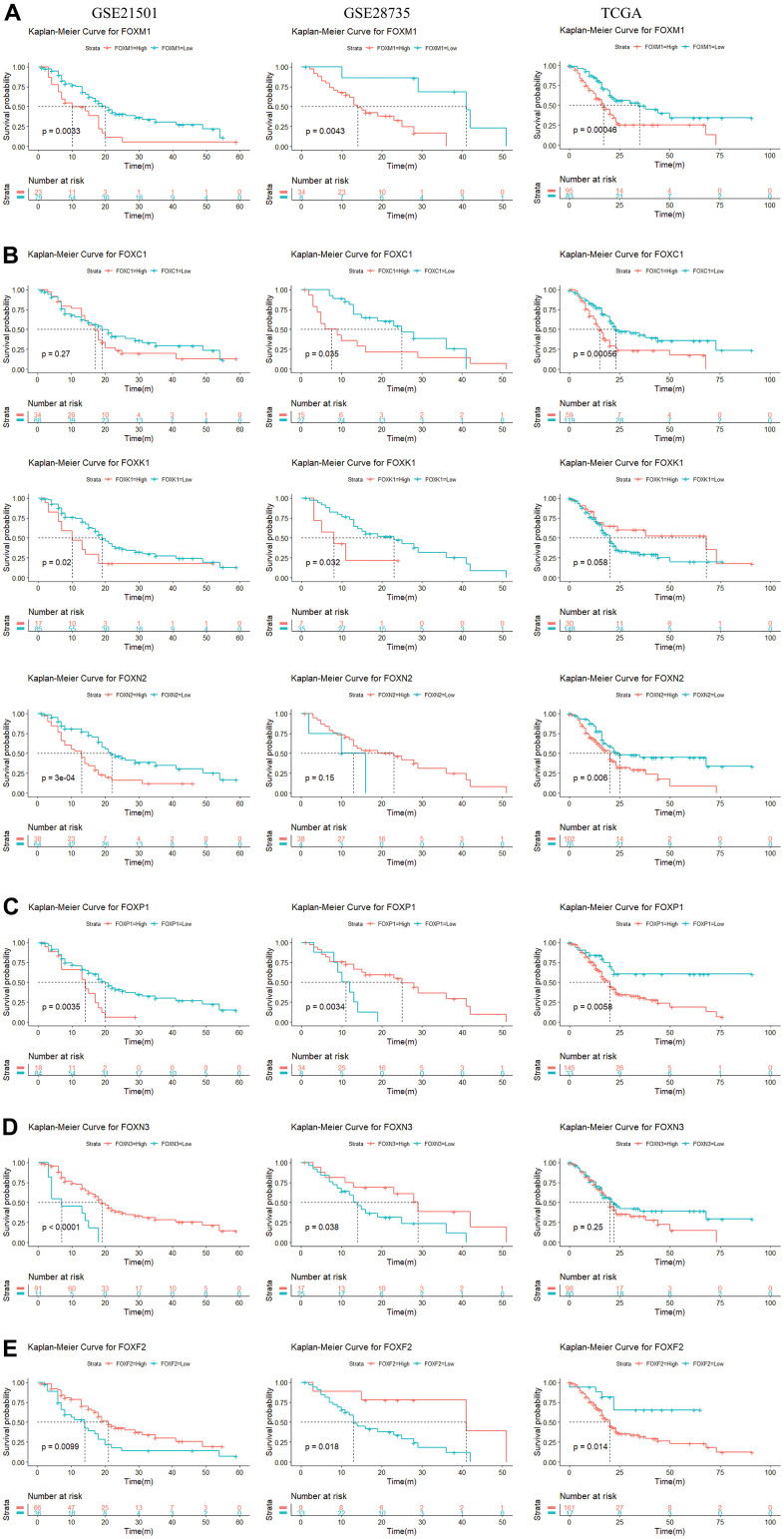
**Survival analysis of 15 differentially expressed FOXs in PAAD patients (GSE21501, GSE28735, and TCGA).** (**A**) High expression of FOXM1 was correlated with short OS in all three datasets. (**B**) High expression of FOXC1, FOXK1, and FOXN2 was negatively correlated with OS in two of three data sets. (**C**) High levels of FOXP1 were related to short OS in the TCGA and GSE21051 databases, but long disease-free survival (DFS) in the GSE28735 database. (**D**) Expression of FOXN3 was positively correlated with OS in the GSE28735 and GSE21051 databases, but showed no significant relationship in the TCGA database. (**E**) Expression of FOXF2 was positively correlated with OS in the GSE28735 and GSE21051 databases but was negatively related to OS in the TCGA database.

We used the R language to evaluate the prognostic value of the 15 DE-FOXs and the progression of PAAD, the correlations between different FOXs, and clinical outcomes. Overall survival (OS) curves revealed a significant association between high transcriptional levels of FOXM1 and short OS in PAAD patients (Figure 2A) in all three datasets (GSE21501, GSE28735, and TCGA). High expression of FOXC1, FOXK1, and FOXN2 were also negatively correlated with OS in two of three data sets (Figure 2B). FOXC2, FOXD1, FOXF1, FOXQ1, and FOXS1 expression were negatively correlated with OS in TCGA, but were not markedly different in GSE28735 and GSE21051 (data not shown). High levels of FOXP1 were related to short OS in TCGA and GSE21051, whereas a long OS was evident in GSE28735 (Figure 2C).

The expression of FOXN3 was positively correlated with OS according to GSE28735 and GSE21051, whereas no significant relationship was evident in TCGA (Figure 2D). Expression of FOXA1 was positively correlated with OS according to TCGA, but no significant relationship was apparent in GSE28735 and GSE21051 (data not shown). Expression of FOXF2 was positively correlated with OS according to GSE28735 and GSE21051 but was negatively related to OS in TCGA (Figure 2E).

Expression of FOXJ1 and FOXL1 had no relevance to OS in GSE21051 but showed a positive correlation for OS in TCGA and GSE28735 (data not shown). Taken together, the results identified FOXM1, FOXC1, FOXK1, and FOXN2 as potential prognostic markers for PAAD.

### Genetic alteration and promoter methylation analyses of FOXs in PAAD patients

**Figure 3:**
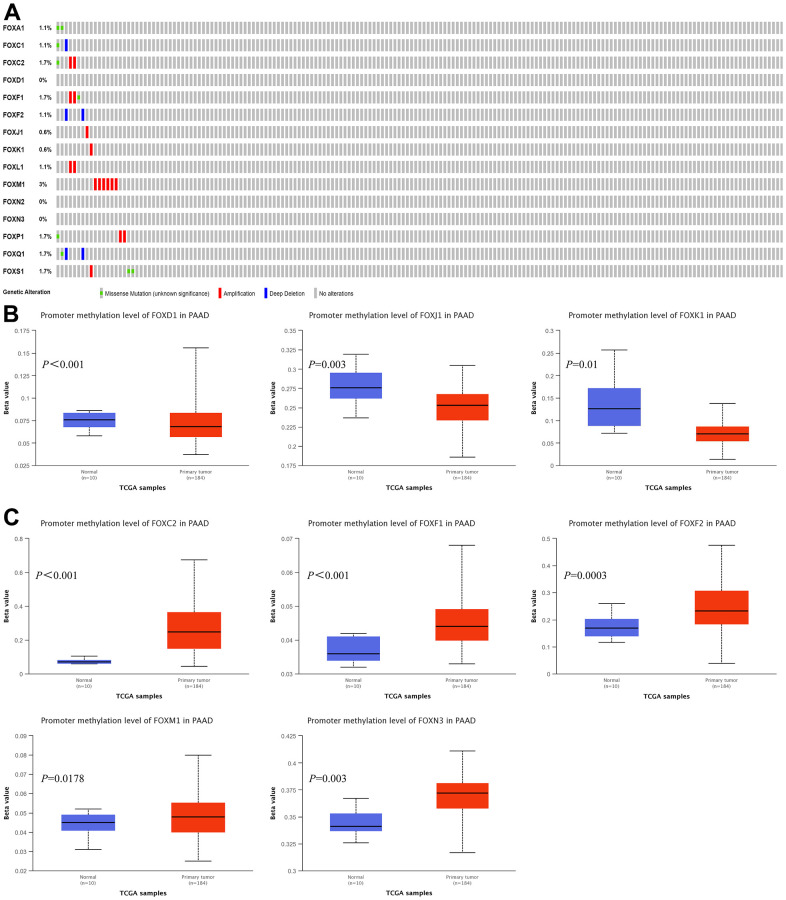
**Genetic alterations and promoter methylation levels of the 15 differentially expressed FOXs in PAAD patients.** (**A**) Summary of genetic alterations of different expressed FOXs in PAAD. All FOXs were altered in 19 samples of 175 patients with PAAD, accounting for 11% of the queried patients/samples. (**B**) Results from UALCAN showed downregulation of the promoter methylation level of FOXD1, FOXJ1, and FOXK1 in tumor tissues. (**C**) The methylation levels of FOXC2, FOXF1, FOXF2, FOXM1, and FOXN3 were upregulated in tumor tissues.

We explored the possibility that the increased FOX expression was due to genomic changes by analyzing the genetic alterations of FOXs in PAAD patients using the cBioPortal. The queried genes were altered in 19 (11%) of the queried patients/samples. FOXM1 was altered in 3% of the queried samples, while FOXC2, FOXF1, FOXP1, FOXQ1, and FOXS1 were altered in 1.7% of the queried PAAD samples. FOXA1, FOXC1, FOXF2, FOXL1 were altered in 1.1% of the samples, while FOXJ1 and FOXK1 were altered in 0.6% of the queried PAAD samples. FOXD1, FOXN2, and FOXN3 showed no genetic alterations in PAAD patients (Figure 3A).

Analysis of the promoter methylation levels of the 15 DE-FOXs by UALCAN revealed downregulation of the promoter methylation levels of FOXD1, FOXJ1, and FOXK1 in tumor tissues (Figure 3B). However, the upregulated methylation levels of FOXC2, FOXF1, FOXF2, FOXM1, and FOXN3 (Figure 3C) indicated that an epigenetic mechanism was not the main reason for the elevated FOX expression in tumor tissues. Promoter methylation of other FOXs showed no significant differences between normal tissues and tumors (data not shown).

### PPI network and gene function analyses of FOXs in PAAD patients

**Figure 4:**
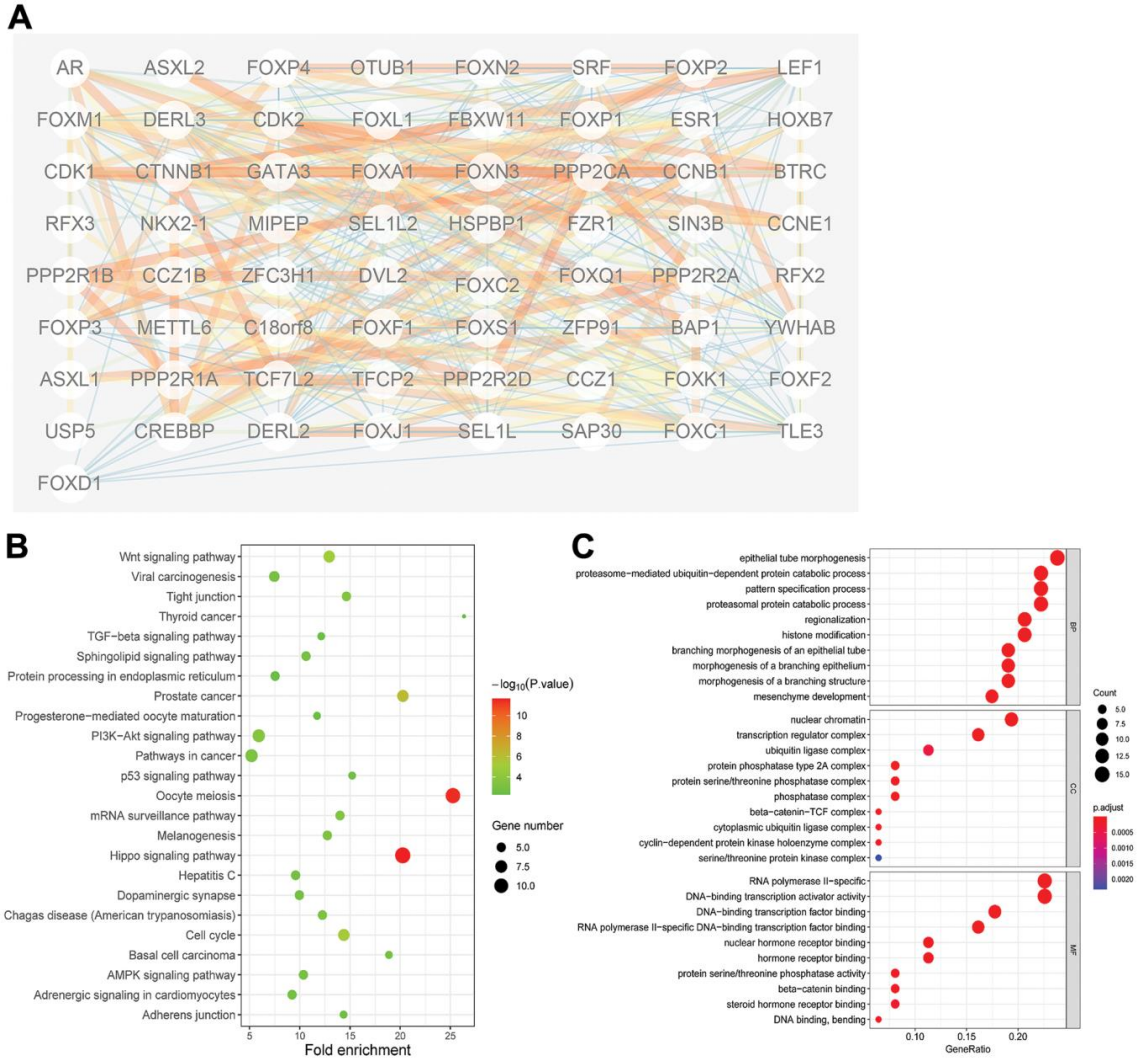
**Protein-protein interaction (PPI) and gene function analyses of the 15 differentially expressed FOXs and related interactors in PAAD patients.** (**A**) STRING analysis of 15 differentially expressed FOXs and 50 interaction proteins. Network nodes represent proteins. Edges represent protein–protein associations, which include known interactions, predicted interactions, and others. PPI enrichment *p*< 0.001. (**B**) All KEGG pathway enrichment results for the 15 FOXs and 50 interaction proteins. (**C**) All GO enrichment analysis results for the 15 FOXs and 50 interaction proteins. MF: Molecular Function; CC: Cellular Component; BP: Biological Process.

PPI network analysis of the differently expressed FOXs with STRING, with the number of proteins that interact with the 15 differently expressed FOXs set to 50, revealed complex interactions among the genes, with 52 nodes and 81 edges (Figure 4A). The interaction score ≥0.7 was considered to indicate high confidence. The following genes showed high connectivity: FOXP2(0.87), AR (0.838), BAP1(0.807), PPP2R2A (0.753), and FOXP3(0.743).

The 50 proteins and DE-FOXs were also subjected to KEGG analysis. The DE-FOXs showed the strongest correlations with oocyte meiosis and the hippo signaling pathway (Figure 4B). The GO results also revealed an association between the functions of the FOXs and their related proteins and epithelial tube morphogenesis, nuclear chromatin, and DNA-binding transcription factor binding. (Figure 4C).

### Immune cell infiltration of FOXs in PAAD patients

**Figure 5:**
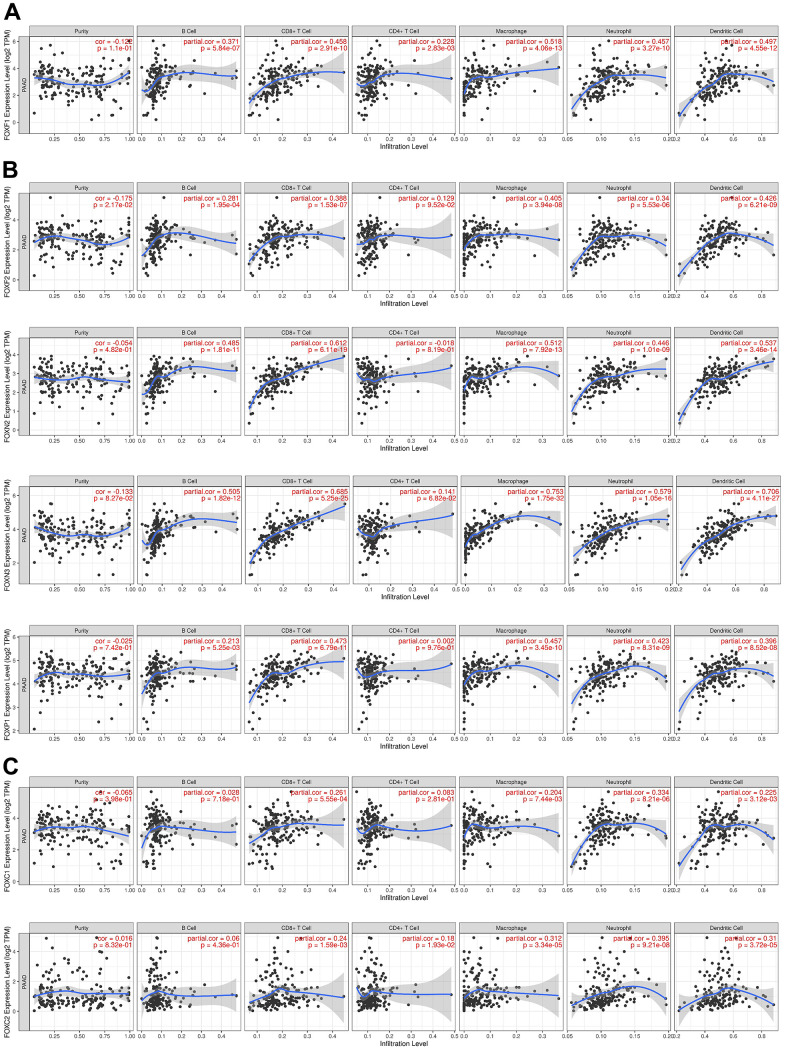
**Correlations between FOX expression and immune cell infiltration.** (**A**–**C**) The expression correlation between distinct FOXs and immune cells was analyzed by TIMER. *p* value is shown in the figures. Partial.cor, purity-corrected partial Spearman’s rho value.

The immune cell level is associated with the proliferation and progression of cancer cells. Exploration of the correlation between the 15 DE-FOXs and immune cell infiltration using the TIMER database showed that a positive correlation between FOXF1 expression and the infiltration of B cells, CD8+ T cells, CD4+ T cells, macrophages, neutrophils, and dendritic cells in PAAD (Figure 5A). Expression of FOXF2, FOXN2, FOXN3, and FOXP1 was positively associated with the infiltration of B cells, CD8+ T cells, macrophages, neutrophils, and dendritic cells in PAAD (Figure 5B). FOXC1 and FOXC2 expression was positively correlated with infiltration of CD8+ T cells, macrophages, neutrophils, and dendritic cells (Figure 5C). FOXK1, FOXM1, and FOXS1 expression was only positively correlated with infiltration of CD4+ T cells (data not shown). FOXQ1 expression was only positively correlated with macrophage infiltration (data not shown). The expression of FOXA1, FOXJ1, and FOXL1 was not related to immunity (data not shown).

### Correlation between FOXs and tumor senescence

**Figure 6:**
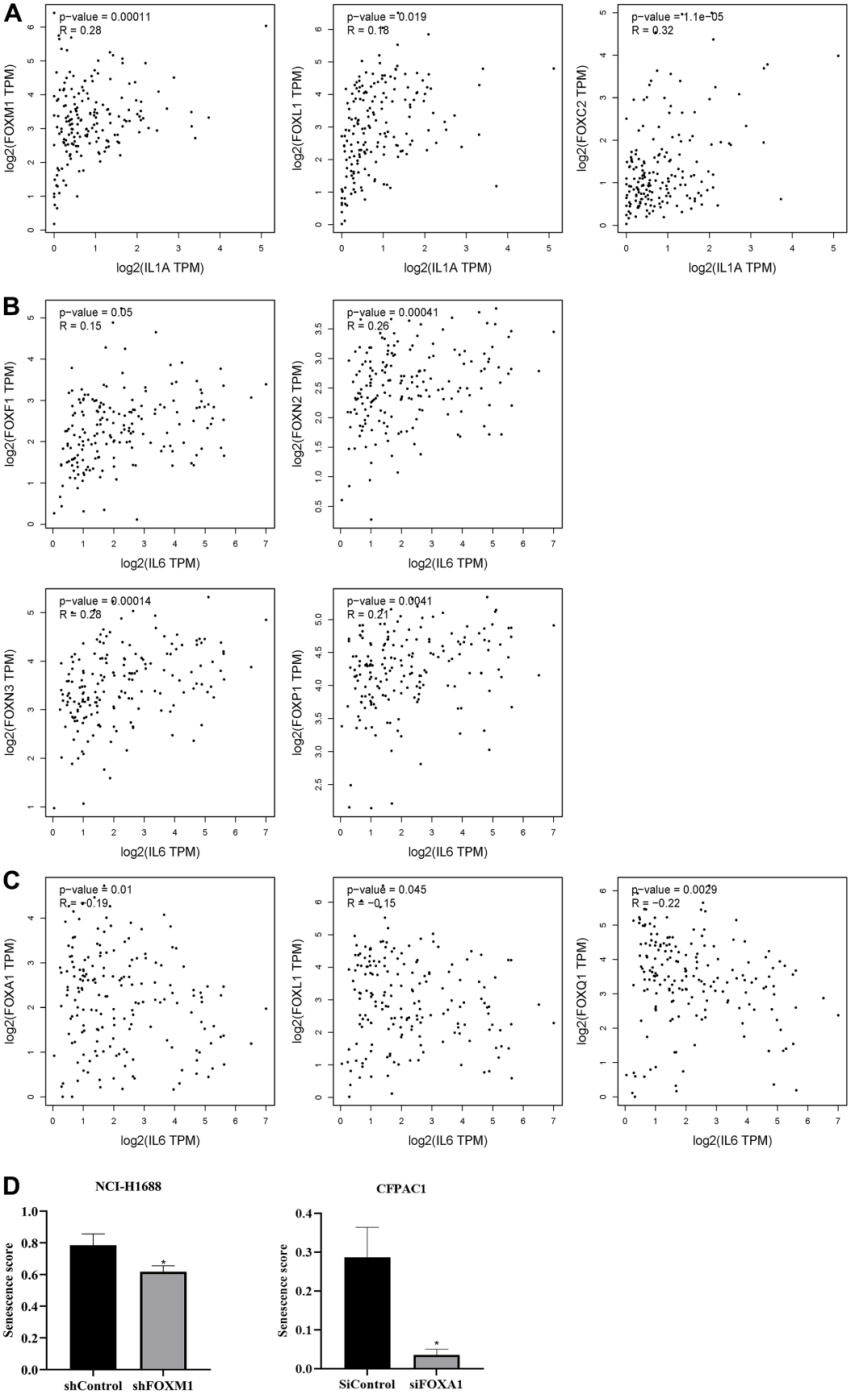
**Correlation between differentially expressed FOXs and tumor senescence.** (**A**) Positive correlation between FOXs and IL-1α in pancreatic adenocarcinoma (PAAD), n=179. (**B**) Positive correlation between FOXs and IL-6 in PAAD, n=179. (**C**) Negative correlation between FOXs and IL-6 in PAAD, n=179. (**D**) Senescence score of FOXM1 and FOXA1 calculated using SENESCopedia. *, *p* < 0.01.

The SASPs are a group of senescence markers that includes pro-inflammatory cytokines, chemokines, growth factors, and matrix remodeling enzymes secreted by senescent cells. Two known SASPs associated with PAAD are IL-1α and IL-6. Analysis of FOXs and IL-1α and IL-6 in PAAD using the correlation module of GEPIA revealed positive correlations between FOXC2, FOXL1 and FOXM1 expression and IL-1α levels, but no other significant correlations (Figure 6A). FOXF1, FOXN2, FOXN3, and FOXP1 expression was positively related to IL-6 levels, while FOXA1, FOXL1, and FOXQ1 expression was negatively correlated with IL-6 (Figure 6B, 6C).

We also used the Cancer SENESCopedia, a gene expression classifier for the detection of senescence in cancer cell samples, to determine the influence of FOXs on senescence. Gene expression data were obtained from GEO with the indicated accession numbers. We used FOX gene, cancer, and RNA-seq as search keywords to preferentially select RNA-seq data that interfered with or promoted overexpression of FOX genes and that showed three duplicates in pancreatic cancer. We obtained one GEO record in pancreatic ductal adenocarcinoma cells (GSE119931 in CFPAC1 cells) and one in lung cancer cells (GSE174462 in lung cancer cell NCI-H1688). Then we processed and analyzed the data using SENESCopedia. As shown in Figure 6D, the senescence score was decreased in the group that interfered with FOXM1, confirming the positive correlation between FOXM1 and IL-1α. However, siFOXA1 also downregulated the senescence score, which contradicted the negative correlation between FOXA1 and IL-6 in Figure 6C.

### Validation of the prognostic role of FOXs in PAAD tissues from patients

**Figure 7:**
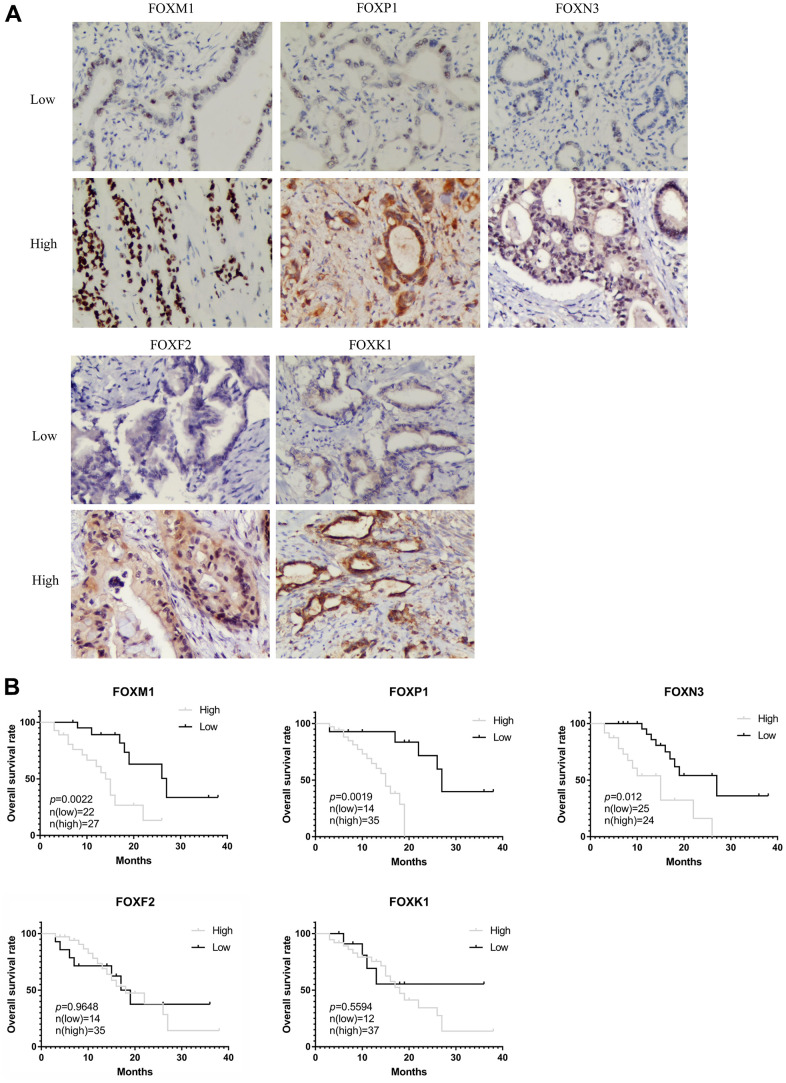
**Validation of the prognostic role of FOXs in PAAD patient tissues.** (**A**) Representative immunohistochemistry (IHC) images (200X) in PAAD tissues with low or high FOXM1, FOXP1, FOXN3, FOXF2, and FOXK1 protein levels. (**B**) Expression of FOXM1, FOXP1, and FOXN3 was negatively correlated with OS in PAAD patient pathology samples. Expression of FOXF2 and FOXK1 had no significant correlation with OS.

We also analyzed the correlation between clinical pathological variables and the protein levels of FOXM1, FOXP1, FOXN3, FOXF2, and FOXK1 in PAAD tissues. Figure 7A shows representative IHC staining images, and the relationship between the clinical pathological characteristics and protein expression are summarized in Table 1. The protein expression level of FOXM1 and FOXK1 was negatively related to venous invasion (p=0.0124, p=0.0254), while the expression level of FOXP1 was positively correlated with histological grade (p=0.0028), pathologic lymph node status (p=0.0098), pathologic tumor status (p=0.0104) and tumor size (p=0.0298). The protein level of FOXN3 was positively correlated with histological grade (p=0.0402), tumor size (p< 0.0001), and pathologic tumor status (p< 0.0001). The overall survival (OS) analysis, as shown in Figure 7B, revealed patients with high FOXM1, FOXP1, and FOXN3 protein expression has shorter OS than patients with low protein expression, whereas the expression of FOXF2 and FOXK1 showed no significant correlation with OS. Those results demonstrated a potential prognostic significance for FOXM1, FOXP1 and FOXN3 in clinical patients.

**Table 1:** Univariate analysis of the protein level and clinicopathological characteristics.

**Clinicopathological features**	**Case**	**FOXM1**			**FOXP1**			**FOXN3**			**FOXF2**			**FOXK1**		
		**High**	**Low**	***P* value**	**High**	**Low**	***P* value**	**High**	**Low**	***P* value**	**High**	**Low**	***P* value**	**High**	**Low**	***P* value**
**Age(years)**																
≤65	22	13	9	>0.9999	14	8	0.2785	9	13	0.3077	14	8	0.2758	17	5	0.2590
>65	27	14	13		21	6		15	12		21	6		20	7	
**gender**																
male	29	18	11	0.1040	21	8	0.8541	12	17	0.2000	22	7	0.4081	20	9	0.1995
female	20	9	11		14	6		12	8		13	7		17	3	
**histological grade**																
I	22	10	12	0.2593	12	10	0.0028	7	15	0.0402	19	3	0.1078	16	6	0.8218
II	24	16	8		23	4		15	9		14	10		19	5	
IV	3	1	2		0	3		2	1		2	1		2	1	
**tumor size**																
<3	23	11	12	0.3965	13	10	0.0298	3	20	<0.0001	14	9	0.1238	17	6	>0.9999
≥3	26	16	10		22	4		21	5		21	5		20	6	
**differentiation degree**																
low	39	22	17	0.7162	27	12		17	22	0.5012	28	11	0.9108	28	11	0.2323
high	10	5	5		8	2	0.3078	7	3		7	3		9	1	
**Pathologic tumor status**																
T1	14	7	7	0.8221	12	2	0.0104	0	14	<0.0001	10	4	0.6092	9	5	0.4864
T2	22	11	11		11	11		14	8		17	5		18	4	
T3	13	9	4		12	1		10	3		8	5		10	3	
**Pathologic node status**																
N0	36	19	17	0.5862	22	14	0.0098	17	19	0.6822	28	8	0.1016	26	10	0.3731
N1	13	8	5		13	0		7	6		7	6		11	2	
**nerve plexus invasion**																
Negative	29	17	12	0.5510	22	7	0.5237	13	16	0.4839	21	8	0.8541	23	6	0.4563
Positive	20	10	10		13	7		11	9		14	6		14	6	
**Venous invasion**																
Negative	16	5	11	0.0124	11	5	>0.9999	6	10	0.0651	11	5	0.4940	14	2	0.0254
Positive	6	6	0		4	2		1	5		5	1		2	4	
Missing	27	16	11		20	7		17	10		19	8		21	6	

## DISCUSSION

PAAD is one of the most aggressive digestive tract tumors, with a 5-year overall survival of 9% [8]. Surgical removal remains the only cure for PAAD, but most patients are diagnosed at an advanced stage or with metastatic tumors, and this limits the usefulness of surgical intervention. For this reason, chemotherapy is often used to improve patient outcomes, but drug-tolerance issues limit the survival benefits [9]. Therefore, seeking new therapeutic targets may provide an opportunity to enhance the efficacy of standard chemotherapeutics or to overcome drug resistance. In the present study, we comprehensively analyzed the expression, prognostic value, mutation, and immune cell infiltration effects of FOXs in PAAD.

We identified seven DE-FOXs (FOXM1, FOXC1, FOXK1, FOXN2, FOXP1, FOXN3 and FOXF2) that might serve as potential prognostic markers of PAAD (Figure 2A, 2B). Among the seven FOXs, we chose five to validate the bioinformatics results in PAAD tissues that we collected. We found that FOXM1, FOXP1, and FOXN3 were negatively correlated with OS and that FOXP1 and FOXN3 were positively correlated with histological grade and tumor size (Figure 7 and Table 1). These results identified the expression of FOXM1, FOXP1, and FOXN3 as possibly important prognostic markers for PPAD.

Contradicting our results, previous studies have shown that FOXN2 may serve as a tumor suppressor in breast cancer [10], lung cancer [11], and human oral cancer [12]. The degradation of FOXN2 promotes tumorigenesis and radioresistance in lung cancer cells [11], but our results identified FOXN2 as a possible oncogene in PAAD (Figures 1B, 2B). Organizational differences may explain this paradox, as certain tumor suppressors are capable turnoff becoming oncogenic depending on the cell context. A well-known example is p53. The wild type p53 is an authentic tumor suppressor; however, mutations confer new functions to p53 and transform p53 into a potent oncogene [13]. Libing Shen et al. have also identified a series of genes that exhibit dual biological functions in 12 cancer types (e.g., they can act as tumor suppressor or oncogenes) [14]. FOXN2 could possibly be an as yet undiscovered gene of this type. Further study and experimental results are needed to confirm a dual function and the underlying mechanism of FOXN2 in PAAD.

Tumor-infiltrating immune cells are found in almost all cancers (including PAAD), and while they usually cannot eliminate tumors [15], they can promote cancer progression by changing the tumor microenvironment. Immune cells account for nearly half of the pancreatic tissue [16], and these immune cells promote tumor progression in various ways. For example, macrophages, located at the invasive front of the tumor, promote the formation of PAAD connective tissue and metastasis of cancer cells through the trend factor PDGF-BB [17].

We also showed that 12 of the 15 DE-FOXs identified in this study are associated with immune infiltration, suggesting that FOXs may be involved in tumor immunity. In fact, a few studies have suggested a role for FOX family members in immunity. For example, FOXA1 overexpression can suppress the cancer immune response and promote cancer immuno- and chemotherapy resistance [18]. FOXC1 mediates the high expression of an oncogenesis long intergenic non-coding RNA 00301, which increases regulatory T cell (Treg) infiltration while decreasing CD8+ T cell infiltration in non-small cell lung cancers [19]. The findings of the present study might reveal detailed immunization information to improve the understanding of the potential roles of FOXs in immunity and provide new perspectives on immunotherapy for PAAD. The relationship between other FOX members and immunity needs further confirmation.

Pancreatic cancer is an inflammation-driven disease, and senescent cells are inextricably linked to the production of inflammation. Aging promotes tumor growth and proliferation by remodeling the tumor microenvironment by promoting the secretion of pro-inflammatory cytokines, chemokines, growth factors, and matrix remodeling enzymes (collectively referred to as SASPs) [20]. The expression of IL-1α is considered to be the initiating event of SASP secretion by senescent cells, while IL-6 is a SASP required for Ras tumor progression. The frequency of RAS mutations in pancreatic carcinoma is high [21]; therefore, although many SASP were found in PAAD, we chose IL-1α and IL-6 for further analysis.

Our analysis of correlations between FOX gene expression and IL-1α or IL6 revealed 10 members that might be involved in tumor senescence. Among these correlations, the positive correlation between FOXM1 and senescence was further verified by SenesCOPEDIA (Figure 6D, left panel). However, FOXM1 has been reported to act as a negative regulator of senescence in squamous carcinoma cells [22], breast cancer [23], and gastric cancer [24]. In our results, the relationship between FOXA1 and aging is contradictory (Figure 6C, 6D). Jingyun Wang et al. found that FOXA1 could induce endometrial cancer cell senescence by interacting with p16INK4a [25]. The true relationship between FOXs and senescence in PAAD still needs further experimental validation.

Our study had some limitations as the results were based on database comparisons and lacked the support of *in vitro* and *in vivo* experimental results. Further exploration is still needed to clarify the mechanism, molecular interactions, clinical applications, and roles of distinct FOXs in immunity or senescence in PAAD.

## MATERIALS AND METHODS

### Data acquisition and bioinformatics analysis

Expression profiles and clinical characteristics of the TCGA (The Cancer Genome Atlas) database were obtained from GEPIA [26] (http://gepia.cancer-pku.cn/index.html) and the GEO (Gene Expression Omnibus) database (http://www.ncbi.nlm.nih.gov/geo/). The overall survival analysis was conducted using the R language. We used the R package “survival” and “survminer” to determine the best cut-off value of the survival curve, and we plotted the Kaplan-Meier survival rate curve in the TCGA, GSE28735, and GSE21501 data sets. The promoter methylation analysis was conducted by UALCAN [27] (http://ualcan.path.uab.edu/index.html).

### Gene expression profiling interactive analysis (GEPIA)

GEPIA is a newly developed analytical tool that uses a standard processing pipeline and consists of data from thousands of tumors and normal tissue samples [26]. We performed differential gene expression analysis to allow the comparison of tumor and normal tissues, pathological stage analysis, and correlative prognostic analysis through GEPIA. Student’s t-test was used to generate a p-value for the expression or pathological stage analysis.

### PPI network and gene function analysis

We obtained 15 FOX family gene binding proteins based on the STRING website [28] (https://string-db.org/). We then downloaded the protein-protein interaction (PPI) data in .tsv format and analyzed it using Cytoscape (Version: 3.9.0) software in the JAVA platform, followed by Kyoto Encyclopedia of Genes and Genomes (KEGG) analysis of 50 proteins and FOX family genes. We uploaded the gene list to DAVID (Database for annotation, visualization, and integrated discovery), and set the selected identifier (“OFFICIAL_GENE_SYMBOL”) and species (“Homo sapiens”), to obtain the data for the function annotation figure. The “tidyr” and “ggplot2” R packages were then used to show the enrichment path. We also used the “clusterProfiler” R package to perform Gene Ontology (GO) enrichment analysis.

### cBioPortal

cBioPortal (https://www.cbioportal.org) is a comprehensive web resource that provides visual and multidimensional cancer genomics data [29]. Data named Pancreatic Adenocarcinoma (TCGA, PanCancer Atlas) were selected for analysis, and we clicked the “Query By Gene” and selected the genomic profiles (mutations, structural variants, and putative copy-number alterations from GISTIC). We entered each gene name in the query box and obtained the genetic alterations of the FOXs.

### Timer

TIMER (https://cistrome.shinyapps.io/timer/) is a user-friendly web interface containing 6 major analytic modules for systematic evaluation of the infiltration of different immune cells and their clinical impact [30]. FOXs were selected as input via the “Gene module” and the generated scatterplots were used to visualize the correlation of FOX expression and immune infiltration levels in PAAD.

### SENESCopedia

The gene expression data obtained from GEO were processed into a tab-separated value file containing RNA sequencing read counts; the first column contained Ensembl gene IDs, and each subsequent column was a sample, with the sample name in the first row and the read counts below. The files were uploaded and senescence scores were calculated by SENESCopedia (https://ccb.nki.nl/publications/cancer-senescence/). GraphPad prism 8 was used to draw graphs and perform statistics.

### Patients

A total of 49 paraffin-embedded pancreatic adenocarcinoma samples collected between 2012 and 2021 from patients with PAAD were obtained from First People’s Hospital of Lianyungang. Clinicopathologic data, including the age, gender, histological grade, tumor size, differentiation degree, pathologic node status, nerve plexus invasion, and venous invasion status were obtained from hospital records. The pathologic metastasis status was excluded from the analysis since only 3 of the 49 patients were diagnosed with metastasis.

### Immunohistochemistry

Wax block slices of pathology samples were placed in a 60° C oven for 2 h, and the three-in-one repair solution was used for dewaxing, hydration, and antigen retrieval, followed by three 5 min washes with phosphate buffered saline (PBS). The primary antibody was added dropwise to the tissue area, at the following dilution ratios: FOXM1 (1:200, 13147-1-AP, Proteintech), FOXF2(1:200, ab198283, abcam), FOXK1(1:400, DF3235, Affinity), FOXP1 (1:400, DF7250, Affinity) and FOXN3 (1:400, bs-13899R, Bioss). The samples were refrigerated at 4° C overnight, then rewarmed for 30 min, washed free of the primary antibody, blocked for10 min, and washed again with PBS. After the secondary antibody incubation, diaminobenzidine (DAB) was added to develop the color and the tissues were counterstained with hematoxylin, dehydrated, sealed with neutral gum after transparency, and observed under a microscope. Immunohistochemical signals were scored by two independent investigators in a double-blind way. The classical semi-quantitative integration method was adopted, and all the results were evaluated based on the numbers of stained cells and the staining intensity. A final score lower than 1 was defined as a low protein level, while a score ≥2 represented a high protein level.

### Statistical analysis

R.4.1.2 and GraphPad Prism 8 were used for statistical analyses. GraphPad prism 8 was also used to draw the survival curve. A two-sided Student’s t-test for unpaired samples was applied to evaluate the significance of the differences in experiments. Pearson correlation coefficients were calculated to measure associations among the mRNA expression level of various genes. The correlation between FOX protein levels and clinical pathological variables was determined by Pearson’s χ2 test. Values of *p* and *q* less than 0.05 were considered statistically significant.
